# Supplementary material for: Precise MRI-histology coregistration of paraffin-embedded tissue with blockface imaging
Source: Imaging Neurosci (Camb). 2025 Aug 8;3:IMAG.a.106. doi: 10.1162/IMAG.a.106 (PMC12336061; doi:10.1162/IMAG.a.106)
Supplement: Supplementary Material [file IMAG.a.106_supp.pdf]

Supplement of

Precise MRI-Histology Coregistration of Paraffin-Embedded Tissue with Blockface Imaging

Yixin Wang[1], William Ho[2], Istvan N. Huszar[3], Phillip DiGiacomo[2], Hossein Moein Taghavi[2], Lee Tao[2], Matthew Choi[2], Nhu Nguyen[2], Samantha Leventis[2], David B. Camarillo[1], Philipp Schlömer[5], Markus Axer[5,6], Wei Shao[7], Mirabela Rusu[2], Inma Cobos[4], Jeff Nirschl[4], Marios Georgiadis[2], Michael Zeineh*[2]

[1] Department of Bioengineering, Stanford University, Stanford, CA, USA

[2] Department of Radiology, Stanford School of Medicine, Stanford, CA, USA

[3] Athinoula A. Martinos Center for Biomedical Imaging, Harvard Medical School, Boston, MA, USA

[4] Department of Pathology, Stanford School of Medicine, Stanford, CA, USA

[5] Institute of Neuroscience and Medicine (INM-1), Forschungszentrum Jülich GmbH, Jülich, Germany.

[6] Department of Physics, School of Mathematics and Natural Sciences, University of Wuppertal, Wuppertal, Germany

[7] Department of Electrical & Computer Engineering, University of Florida, Gainesville, FL, USA

*Corresponding Author

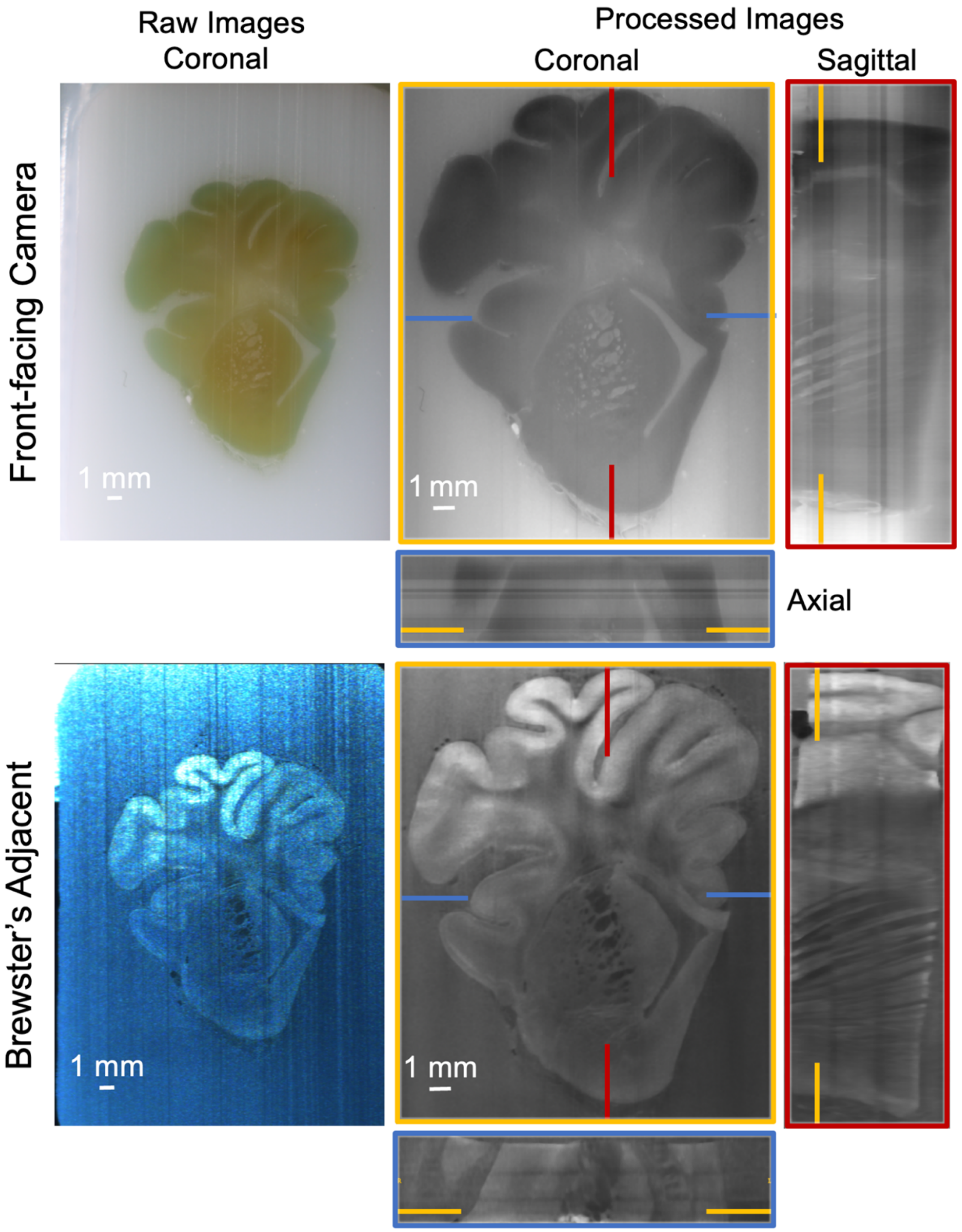

Supplement Figure S1: *Comparison of raw and processed blockface images of the white-paraffin embedded pig coronal slab captured using two different lighting setups, both designed to eliminate depth information: a front-facing camera and a Brewster's angle adjacent camera.*

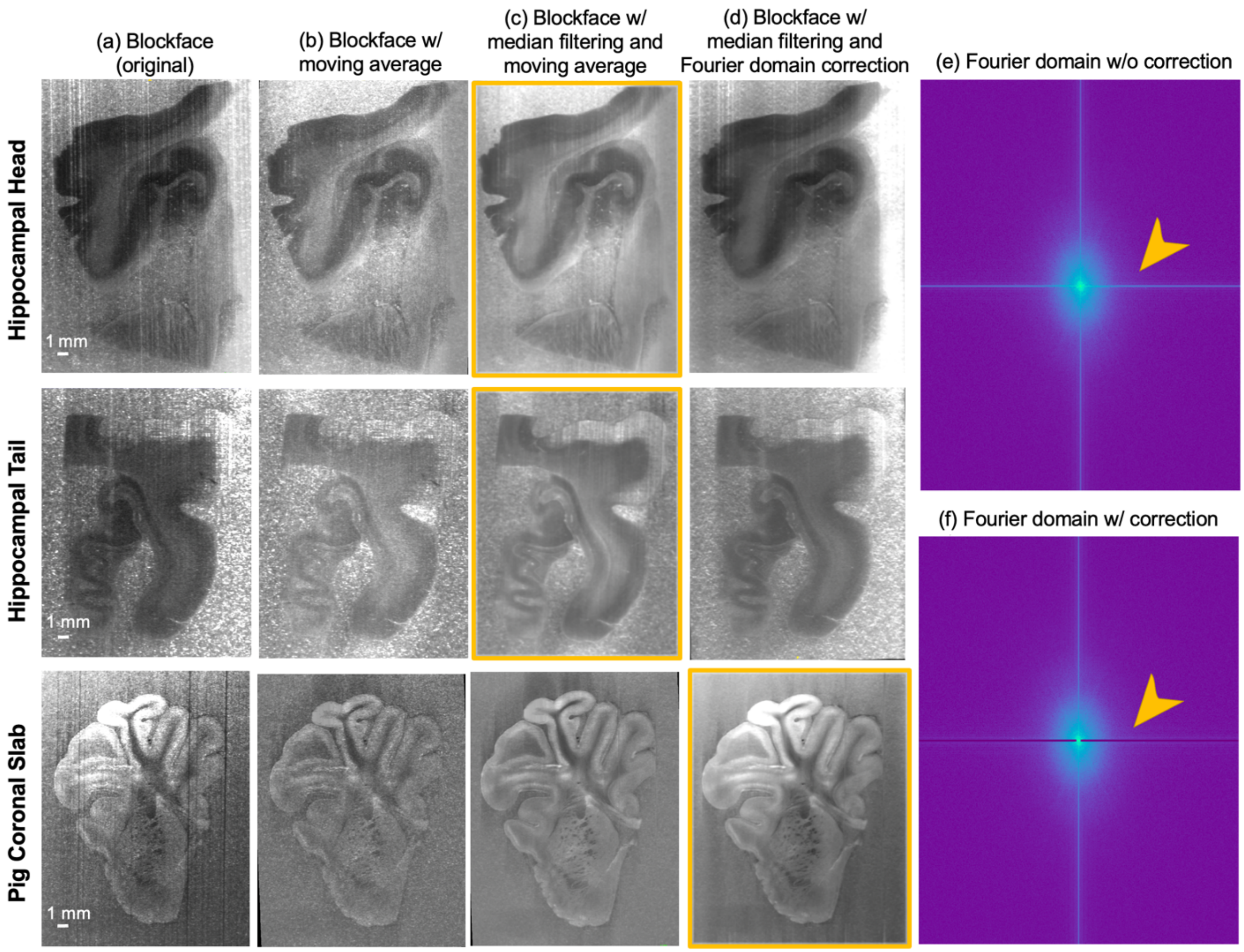

Supplement Figure S2: *Blockface Imaging Processing. (a) Reconstructed and cropped blockface volumes. To address the vertical line artifact from sectioning, (b) a moving average method was applied in the spatial domain (c) with subsequent median filtering, or (d) a Fourier domain correction was adopted, where signals of the line artifacts were filtered out (pointed by orange arrows) in the Fourier domain (e, f). Images within the orange frames were identified as the best quality and selected for subsequent registration with MRI.*

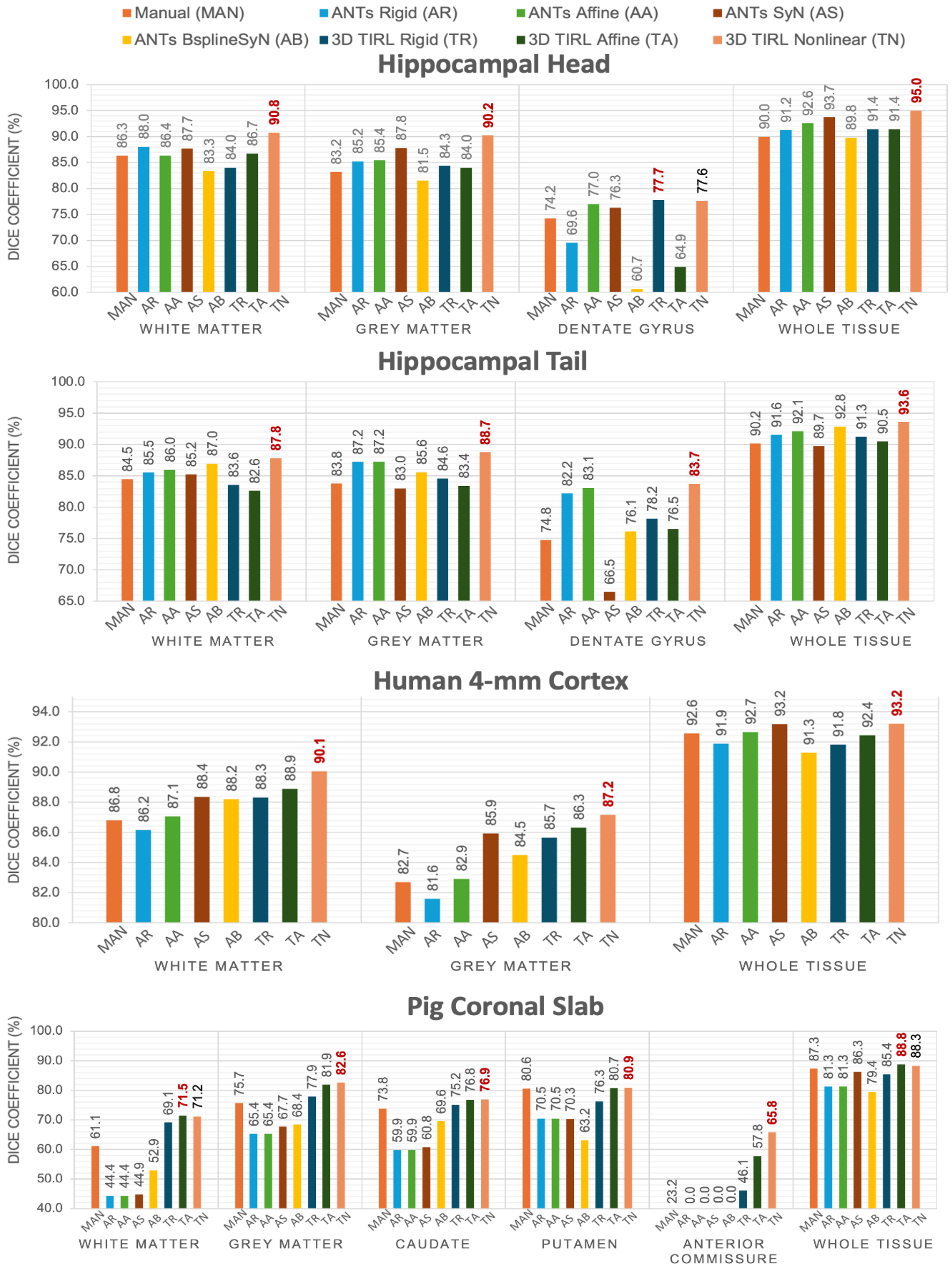

Supplement Figure S3: *Dice similarity coefficient scores (%) of the segmentations of MRI and blockface volumes after coregistration across different specimens. "Whole Tissue" represents the score for the entire composite area. The highest score (best overlap) is indicated in* ***Red****.*

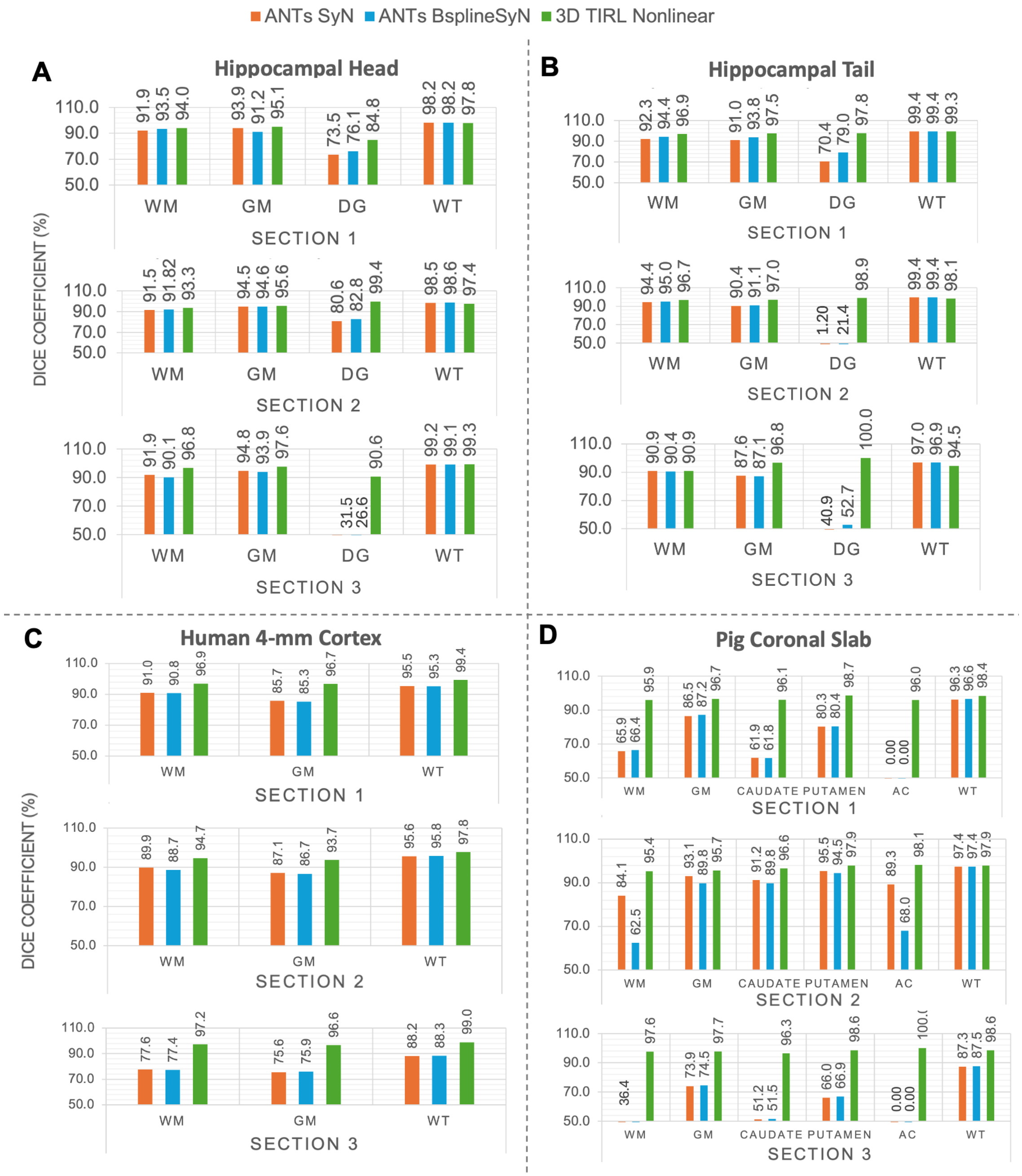

(WM: White Matter, GM: Grey Matter, DG: Dentate Gyrus, AC: Anterior Commissure, WT: Whole Tissue)

Supplement Figure S4: *Dice similarity coefficient (%) analysis for 2D registration of MRI and histology across multiple hippocampal head, tail, 4-mm cortex and pig coronal slab slides with H&E staining. TIRL shows a superior performance in the dentate gyrus (DG) and Anterior Commissure (AC).*

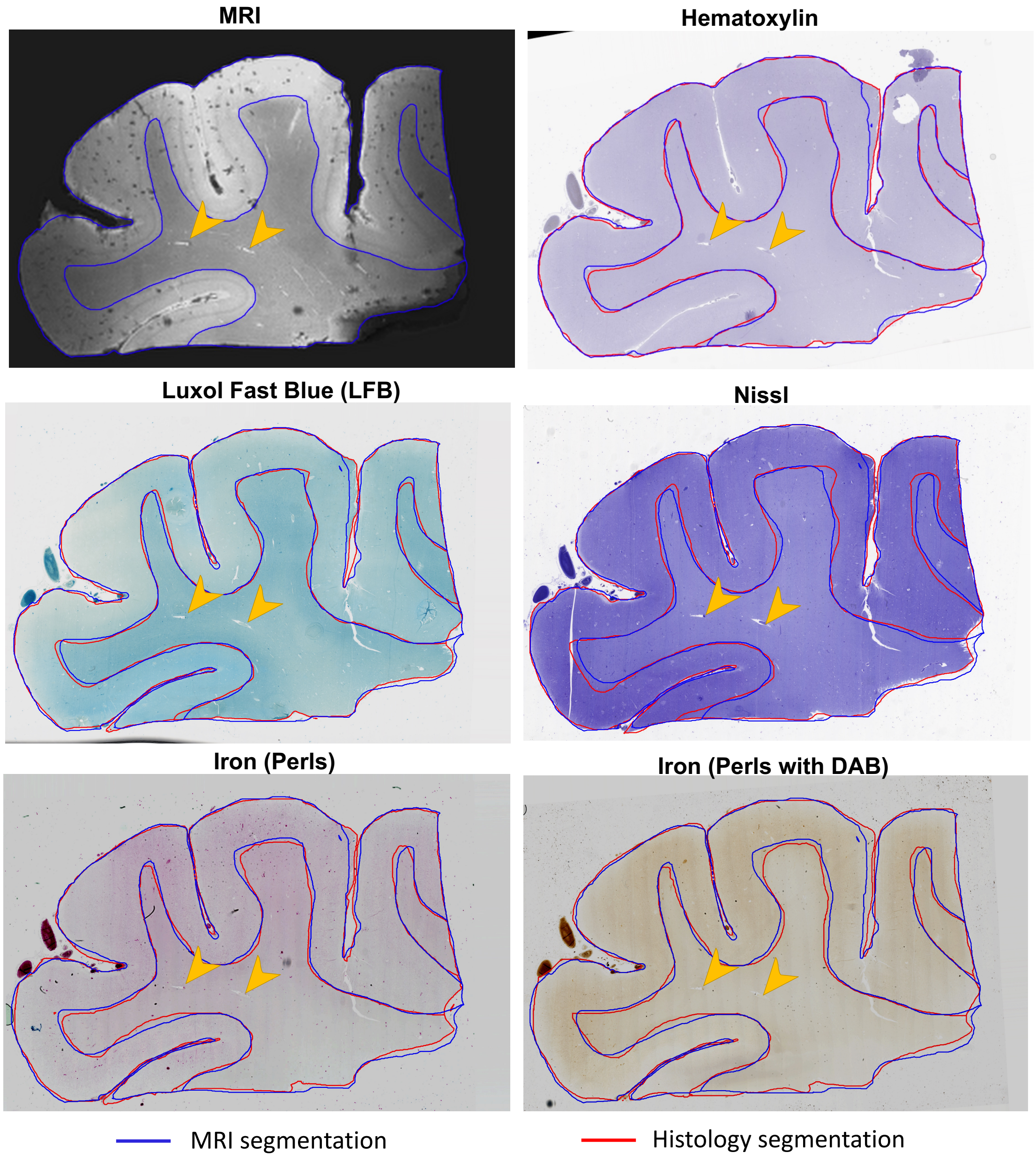

Supplement Figure S5: *Adjacent sections from Human 2-mm Cortex stained with Hematoxylin, Luxol Fast Blue (LFB), Nissl, Iron (Perls), and Iron (Perls with DAB) were shown registered to MRI (top left). White matter and grey matter were manually segmented on MRI slices and histology sections transformed into MRI space. Vessels were marked by orange arrows as internal landmarks. Brightness and contrast were adjusted for Iron (Perls) and Iron (Perls with DAB) to enhance stain visibility in the figure.*

Protocol for Precise Positioning of Camera, Light Source, and Microtome for Blockface Imaging

Overview Setup:

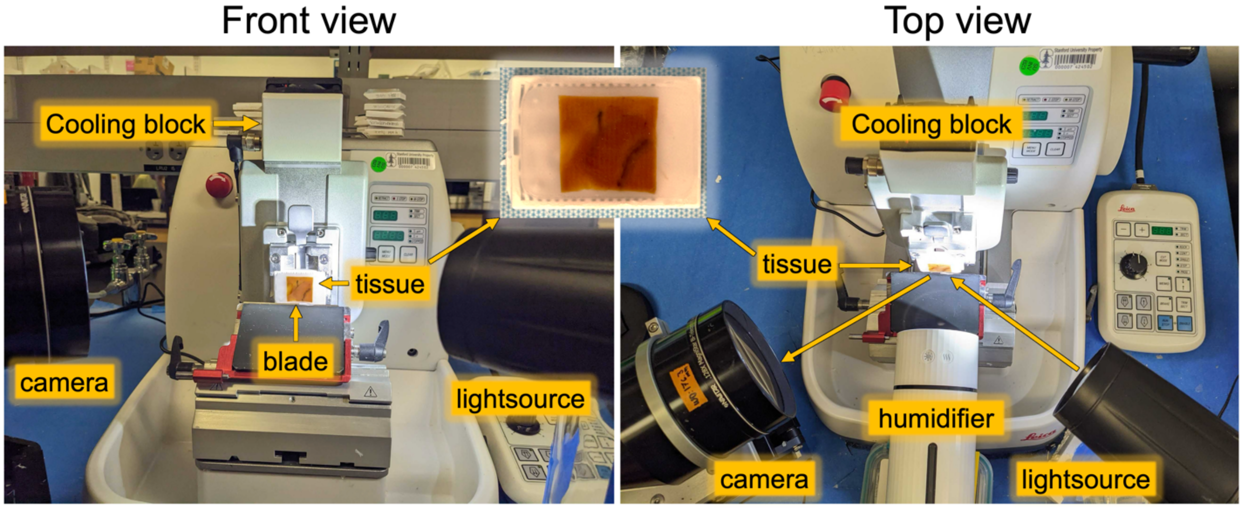

Camera: PL-D775 5.0MP rolling shutter CMOS USB3.0 color camera; Pixellink color sensor: MT9P006; mono sensor: MT9P031, Navitar, Inc.

Bi-telecentric lens: Bi-telecentric 0.0128X F/7 C-MOUNT lens, Magnification: 0.128X, Working Distance: 176.3 mm, Telecentricity: 0.05°, Field Depth: 31 mm, Average Transmittance from 460-630nm: 97%, Navitar, Inc.

Microtome: HistoCore NANOCUT R microtome, Leica, Inc.

Cooling block: Leica RM CoolClamp, Leica, Inc.

Portable humidifier: Palanchy, Inc.

1. Aligning the Microtome

1) Place a metal ruler flush against the backside of the microtome, ensuring it touches the tabletop to create a stable reference area for measurement.
2) Use an additional ruler to measure the distance between the edge of the microtome and the tabletop.
3) Confirm that the measured distance is consistent along the entire microtome edge to ensure proper alignment.

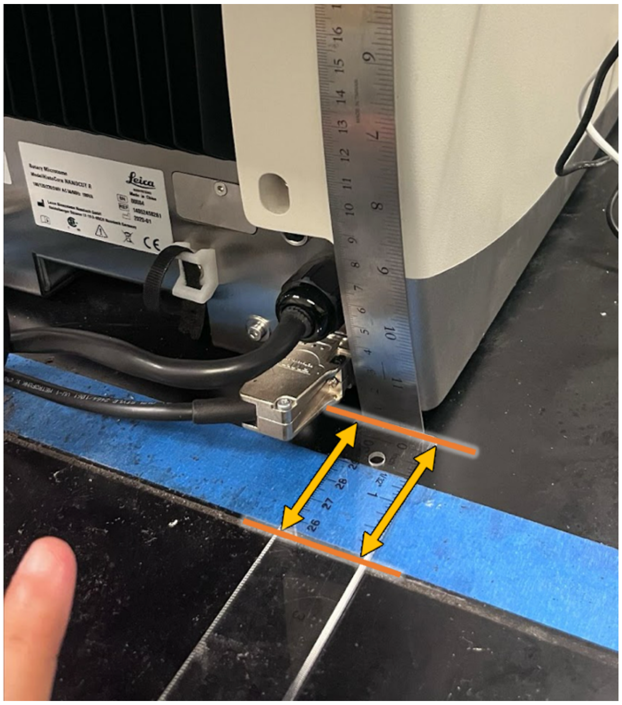

2. Positioning the Knife Holder Base

1) Ensure that the edge of the knife holder base (upper arrow) is perfectly aligned with the edge of the microtome base (lower arrow).

2) Verify alignment visually and through manual measurements for precision.

3) Place the cutting angle at 5.

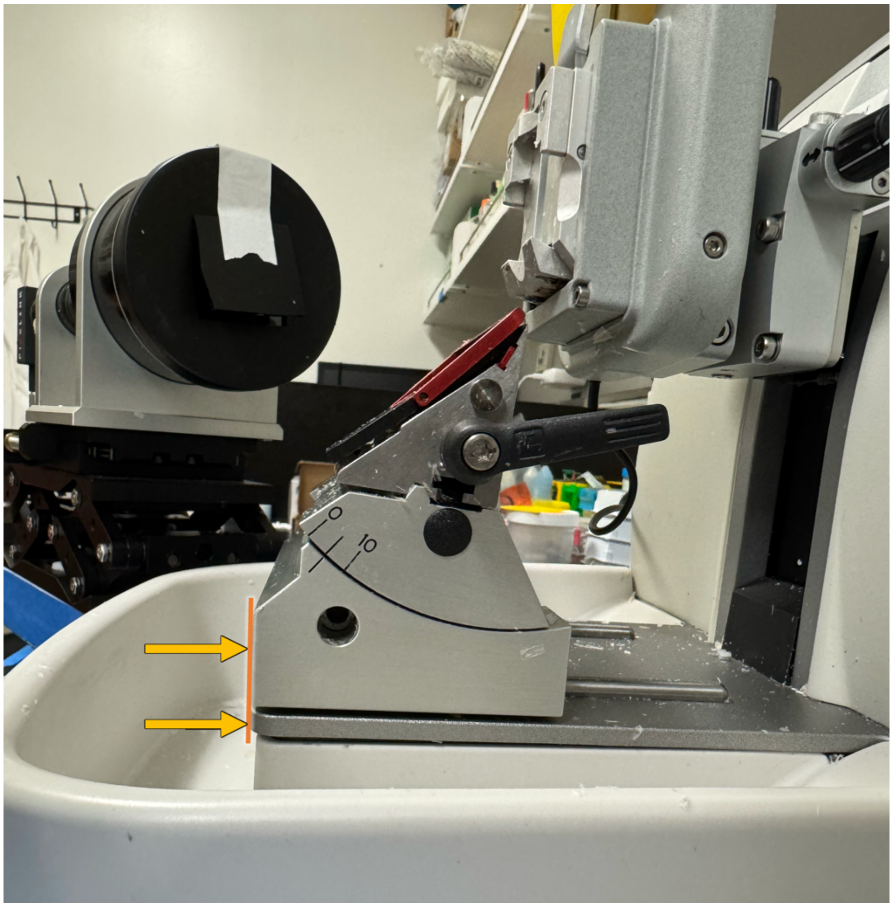

3. Setting the Distance Between the Camera and Microtome

1) Position the block surface in the cutting position before measuring the working distance.
2) Place a ruler horizontally from the block holder lever to the tip of the camera lens cover.
3) Adjust the camera so the working distance between the lens and the middle line of the block surface should follow the working distance of the lens, 17.63 cm.
4) After accounting for the ~2-mm offset between the lens cap and the lens itself, we should measure approximately ~17.5 cm between the lens's front edge and the block surface's middle line.

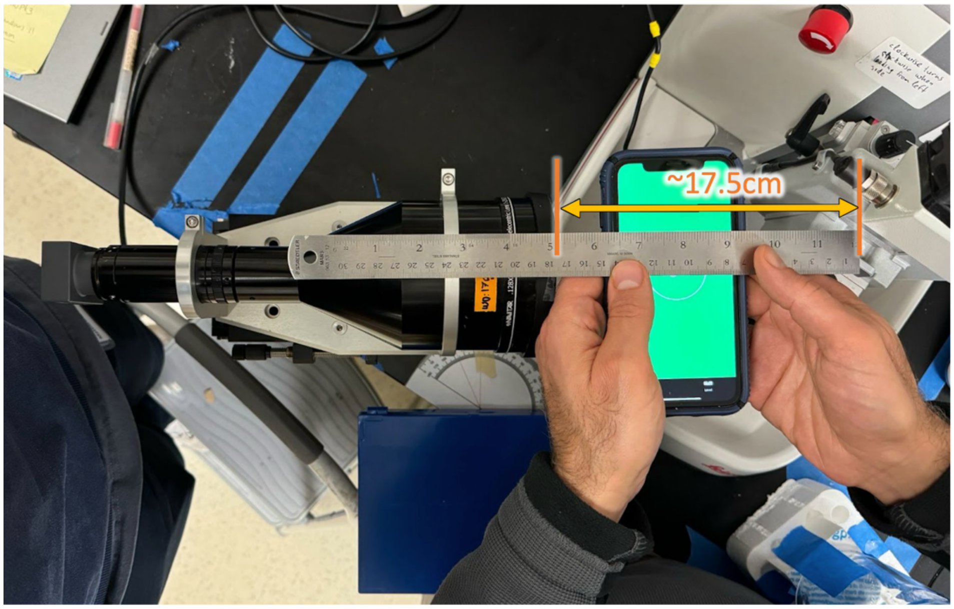

4. Adjusting the Camera Angle

1) The camera should be positioned at Brewster's angle (57° from the normal for the paraffin-air boundary).

2) Place a protractor near the camera to measure the angle between the camera and desk edges, ensuring it is set to 33°.

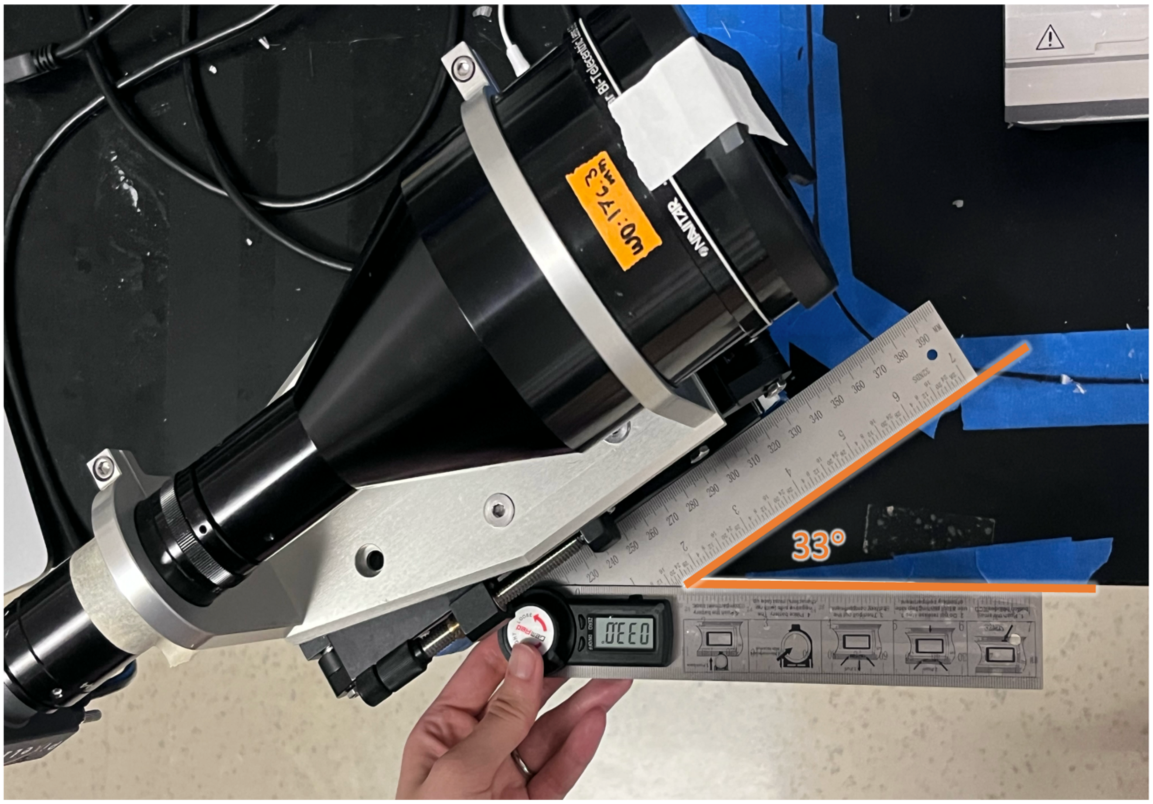

5. Light Source Positioning

1) Setting the Light Source at Brewster's angle
   a. Adjust the light source to maximize brightness on the tissue block surface.
   b. Set the angle between the light source edge and the desk edge to 33°
   c. Identify Brewster's angle by observing the inversion between gray and white matter and very bright reflective surface.
2) Transitioning from Brewster's to the desired Brewster's-Adjacent angle
   a. Increase the horizontal angle from 33° to 36°.
   b. Raise the light source by 2 cm.
   c. Tilt the light source downward by 1°.
   d. Make minor adjustments to ensure homogeneous illumination, the presence of some reflective lines, but not as many as at Brewster's angle.
   e. Secure the light source.
3) Transitioning to Fully Off-Brewster's Angle
   a. Further raise the light source by 5 cm.
   b. Tilt the light source downward by 17°.

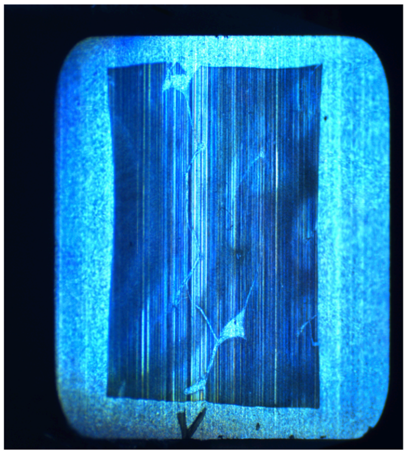

On Brewster's

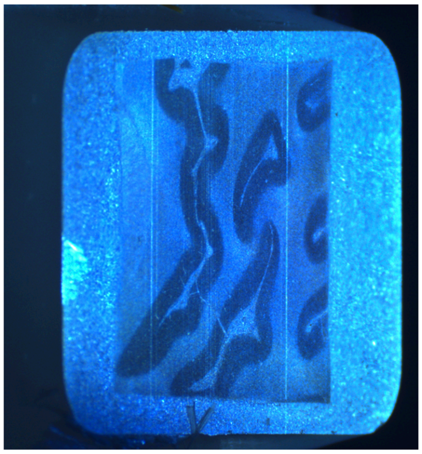

Brewster's Adjacent

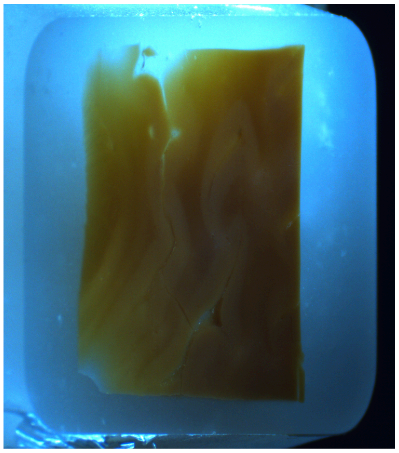

Off Brewster's
